# Risk factors for venous irritation in patients receiving vinorelbine: a retrospective study

**DOI:** 10.1186/s40780-018-0122-2

**Published:** 2018-10-01

**Authors:** Yoshihito Morimoto, Kumika Miyawaki, Reisuke Seki, Kazuhiro Watanabe, Masayoshi Hirohara, Takao Shinohara

**Affiliations:** 1 Education and Research Center for Clinical Pharmacy, Showa Pharmaceutical University, 3-3165 Higashi-Tamagawagakuen, Machida, Tokyo, 194-8543 Japan; 2 Department of Pharmacy, Kyorin University Hospital, 6-20-2 Shinkawa, Mitaka, Tokyo, 181-8611 Japan

**Keywords:** Vinorelbine, Venous irritation, Risk factors, Non-small cell lung cancer, Breast cancer

## Abstract

**Background:**

Vinorelbine is known to be effective in the treatment of non-small cell lung cancer and breast cancer. However, venous irritation is a common side effect. Although there have been some reports on risk factors for venous irritation in patients receiving vinorelbine, the factors evaluated have been limited and the results inconclusive. The aim of this study was to identify risk factors for venous irritation in patients receiving vinorelbine, and factors likely associated with venous irritation, including new factors such as hot compress with a hot towel  for prevention of venous irritation.

**Methods:**

We retrospectively reviewed patients treated with vinorelbine at Kyorin University Hospital, Japan, between March 2013 and December 2016 and divided them into the two groups according to whether or not they had venous irritation. Clinical characteristics were compared between the two groups.

**Results:**

Venous irritation occurred in 24 (38.1%) of 63 patients who received vinorelbine. The median number of times vinorelbine was administered before onset of venous irritation was 3 (range 1–14). The group with venous irritation had a significantly lower body surface area than the group without venous irritation (*p* = 0.035). Low body surface area was also the only significant risk factor for vinorelbine-associated venous irritation in multivariate analysis (adjusted odds ratio 70.42 per 1 m^2^decrement, 95% confidence interval 1.54–3236.25, *p* = 0.029). There was no association between the occurrence of venous irritation and the other covariates, such as use of a hot compress, history of diabetes mellitus, or use of a generic formulation of vinorelbine.

**Conclusion:**

Low body surface area may be a risk factor for venous irritation in patients receiving vinorelbine. Use of hot compress with a hot towel did not prevent venous irritation.

## Background

Venous irritation is one of the discomforting toxicities that can occur with peripheral intravenous therapy. Various drugs can induce venous irritation when administered via peripheral venous infusion, and the osmolality and pH of the solution are reported to influence the likelihood of venous irritation [1]. Moreover, reaction to the drug itself is considered a significant factor in the occurrence of venous irritation. Anticancer drugs are recognized to be strong irritants and often induce venous irritation [2]. Patient factors that affect the extent of extravasation associated with anticancer drugs, the symptoms of which are similar to those of venous irritation, include small and fragile veins, number of previous cycles of chemotherapy, and obesity [3]. Patient-related and drug-related factors work together to influence the risk of developing venous irritation.

Vinorelbine (VNR) is a vinca alkaloid-derived anticancer drug that has antitumor activity against non-small cell lung cancer and breast cancer [4–7]. Although VNR is widely used, venous irritation is a common side effect in patients receiving this drug. The mechanism by which VNR causes venous irritation is reported to be vascular endothelial cell injury due to oxidative stress [8, 9]. Several risk factors for venous irritation caused by VNR have been reported. Yoh et al. found that a high body mass index (≥ 25) was associated with an increased risk of venous irritation [10]. Yamada et al. reported an increased likelihood of venous irritation in patients receiving a VNR dose ≥ 40 mg [11]. However, the parameters evaluated in these studies were limited and the results inconclusive. Retrospective analyses of strategies to prevent venous irritation associated with VNR, including administration of dexamethasone after VNR and bolus injection of VNR, have also been reported [12–14]. One randomized trial found no significant difference in the risk of venous irritation between VNR administered as a 1-min bolus and VNR administered as a 6-min infusion [15]. Application of a hot compress has been reported to protect against venous irritation and injection site reactions [16, 17]. Using a hot pack as a hot compress can cause burns, so we use a hot towel instead, which is less likely to cause thermal injury and is easier to apply.

The aim of this study was to analyze the risk factors for venous irritation in patients receiving VNR, including preventive use of hot compress with a hot towel and use of a generic formulation.

## Methods

This retrospective study involved patients who received administered VNR at Kyorin University Hospital between March 2013 and December 2016. Outcomes were assessed from March 2013 to February 2017. One patient who received both branded and generic formulations of VNR was excluded. The study protocol was approved by the Ethics Committee of Kyorin University Hospital (approval number 858) and was conducted in accordance with the Declaration of Helsinki. The need for informed consent was waived in view of the retrospective and observational nature of the study. An opt-out approach was used with the disclosure of website (URL: http://www.kyorin-u.ac.jp/hospital/clinic/pdf/yakuzaibuekigakutyuousa.pdf).

### Regimen

In all regimens, VNR was administered on days 1 and 8 of each treatment cycle. In the Department of Respiratory Medicine, the usual VNR dose of 25 mg/m^2^ is decreased to 20 mg/m^2^ when combined with cisplatin (CDDP) because radiation therapy is administered concurrently. VNR is usually administered via the main route, but is administered via a side port in this department. VNR was diluted by 50-mL physiologic saline in all patients. After administration of VNR, a 250-mL washout infusion of physiologic saline is administered. On day 1 of each cycle of VNR + CDDP, CDDP was administered following VNR rather than the washout infusion of physiologic saline. Mannitol was administered as a diuretic in all patients who received VNR + CDDP. In March 2016, Kyorin University Hospital switched from the branded formulation of VNR (Navelbine, Kyowa Hakko Kirin Co., Ltd., Tokyo, Japan) to a generic formulation (Rozeus, Nippon Kayaku Co., Ltd., Tokyo, Japan).

### Prevention of venous irritation

A hot towel was applied as a hot compress when VNR was administered in the Department of Thoracic Surgery. The area on the trunk near the administration site was warmed with the hot towel from about 10 min before administration of VNR to the end of the infusion. The hot towel was warmed to about 60°C in a towel steamer (NS-910, Atom Medical Corporation, Tokyo, Japan).

### Evaluation

We collected data on patient characteristics and occurrence of venous irritation and divided the patients into a venous irritation group and a non-venous irritation group. The diagnosis of venous irritation was made by the attending physician. The subspecialty of each attending physician was thoracic surgery, respiratory medicine, or breast surgery. The incidence of venous irritation was recorded from the first day of administration of VNR to the end of the treatment course. The following patient demographic and clinical data were collected from electronic medical and pharmacy records and compared: age, sex (male or female), body surface area, body mass index (≥ 25 or < 25), regimen (single or combination), department (respiratory medicine or surgery), VNR dose (≥ 40 or < 40 mg), route of administration (via side port or main route), drug formulation (branded or generic), history of diabetes mellitus (yes or no), premedication dose of dexamethasone, and use of hot compress with a hot towel (yes or no).

### Statistical analysis

Continuous variables were compared between the group with venous irritation and the group without venous irritation using the Student’s *t*-test or Mann-Whitney *U* test as appropriate. Categorical variables were compared using Fisher’s exact test. Variables with a *p*-value < 0.25 in the univariate analysis were then directly entered into the multivariate logistic regression model. Statistical analysis was performed using EZR version 1.35 software (R Foundation for Statistical Computing, Vienna, Austria) [18]. A *p*-value < 0.05 was considered statistically significant.

## Results

### Patient characteristics

The clinical and demographic characteristics of the 63 patients (41 men, 22 women) who received VNR during the study period are shown in Table 1. No patients treated in the Department of Thoracic Surgery received more than 6 cycles of adjuvant VNR + cisplatin (CDDP) pre- or postoperatively. There were no patients who received fosaprepitant.

**Table 1:** Patient demographics and clinical characteristics (*n* = 63)

Variable	Value
Median age, years (range)	65.0 (35–84)
Sex, male/female	41/22
Median body height, cm (range)	163.8 (144.9–180.9)
Median body weight, kg (range)	59.4 (39.6–80.5)
Median body surface area, m^2^ (range)	1.67 (1.27–1.92)
Median body mass index, kg/m^2^ (range)	22.3 (16.7–29.3)
Chemotherapeutic regimen, (n)	
VNR alone	12
VNR + CDDP	45
VNR + HER	5
VNR + GEM	1
Department	
Respiratory medicine	26
Thoracic surgery	26
Breast surgery	11
Median VNR dose, mg/body (range)	37 (29–47)
Median total cycles, n (range)	4 (1–16)
Administration, main route/side port	19/44
Drug formulation, branded/generic	52/11
History of diabetes mellitus, yes/no	10/53
Dose of dexamethasone used for premedication 13.2/9.9/6.6 mg	38/6/19
Hot compress with hot towel, yes/no	14/49

*VNR* vinorelbine, *CDDP* cisplatin, *HER* trastuzumab, *GEM* gemcitabine

### Venous irritation

**Fig. 1:**
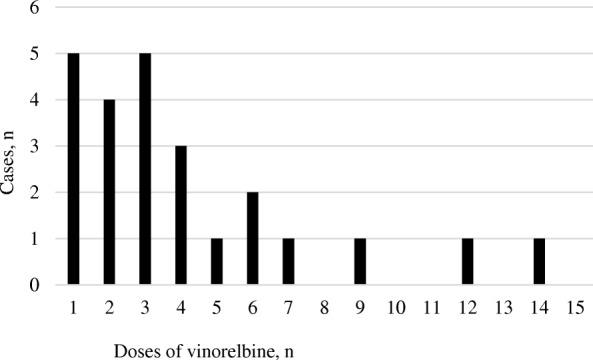
Time of onset of venous irritation (*n* = 24)

Venous irritation occurred in 24 (38.1%) of the 63 patients who received VNR during the study period. Fig. 1 shows the time of onset of venous irritation. The median number of times VNR was administered before onset of venous irritation was 3 (range 1–14) times. In many cases, venous irritation occurred in the early period during treatment.

### Risk factors

**Fig. 2:**
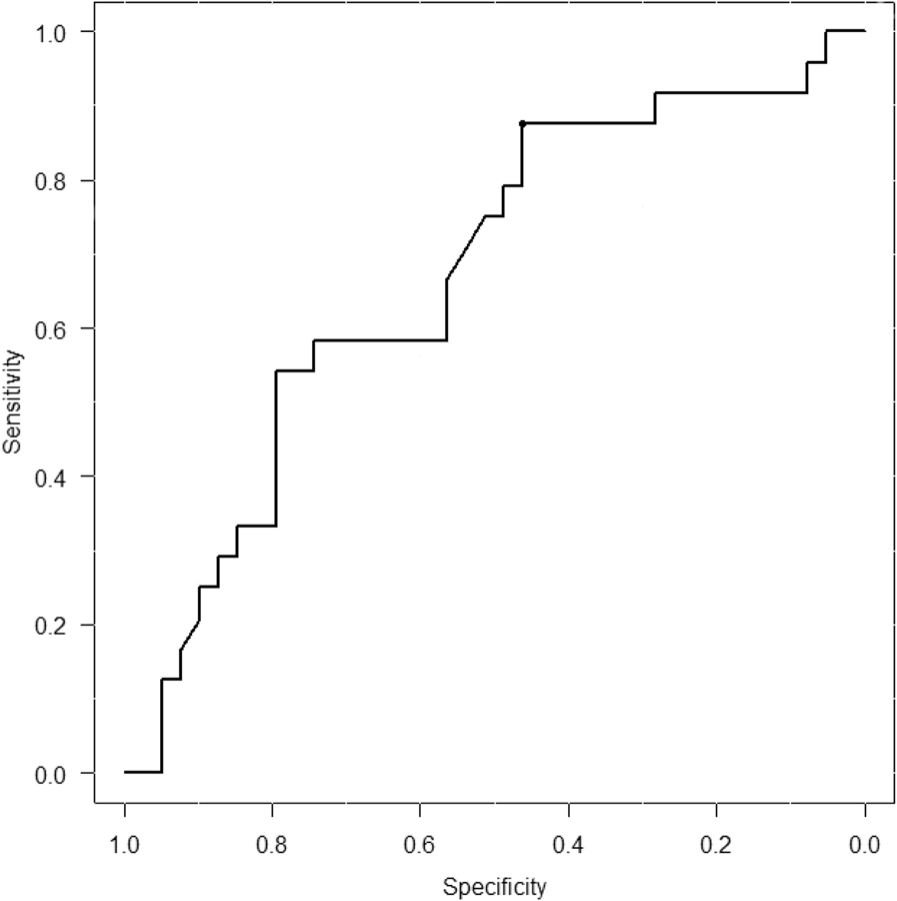
Receiver operating characteristic (ROC) curves for body surface area as a predictor of venous irritation. The area under the ROC curve for body surface area was 0.668. The optimal cutoff value for body surface area was determined to be 1.72 m^2^ (sensitivity 87.5%, specificity 46.2%)

Body surface area was significantly smaller in the group with venous irritation than in the group without venous irritation (*p* = 0.035; Table 2). Patients treated in the Department of Surgery developed venous irritation more often than their counterparts in the Department of Respiratory Medicine, but the difference was not statistically significant (*p* = 0.188). There was no association between likelihood of developing venous irritation and body mass index, VNR dose, use of a side port as the route of administration, use of a generic formulation, or history of diabetes mellitus. Use of hot compress with a hot towel did not prevent venous irritation (*p* = 0.124). When factors with a *p*-value < 0.25 (body surface area, department in which treatment was provided, and application of a hot towel compress) were analyzed in multivariate analysis, only body surface area remained as a statistically significant predictor of the risk for venous irritation (*p* = 0.029; Table 3). We then conducted post-hoc receiver operating characteristic (ROC) curve analysis to evaluate the relationships between the venous irritation onset and the body surface area. The area under the ROC curve for body surface area was 0.668 with a optimal cutoff value of 1.72 m^2^ (Fig. 2).

**Table 2:** Comparison of demographic and clinical characteristics between the group with venous irritation and the group without venous irritation

Variable		Group with venous irritation (*n* = 24)	Group without venous irritation (*n* = 39)	*p*-value
Age, years	Median (range)	64 (35–73)	65 (39–84)	0.514^b^
Sex (%)	Male	14 (58.3)	27 (69.2)	0.423^c^
	Female	10 (41.7)	12 (30.8)	
Body surface area, m^2^	Mean ± SD	1.60 ^a^ ± 0.14	1.68 ^a^ ± 0.16	0.035^d^
Body mass index, n (%)	≥ 25	3 (12.5)	9 (23.1)	0.345^c^
	< 25	21 (87.5)	30 (76.9)	
Regimen (%)	Single	4 (16.7)	8 (20.5)	1^c^
	Combination	20 (83.3)	31 (79.5)	
CDDP administration	Yes	18 (75.0)	27 (69.2)	0.776^c^
	No	6 (25.0)	12 (30.8)	
Department (%)	Respiratory medicine	7 (29.2)	19 (48.7)	0.188^c^
	Surgery	17 (70.8)	20 (51.3)	
VNR dose, mg (%)	≥ 40	6 (25.0)	15 (38.5)	0.410^c^
	< 40	18 (75.0)	24 (61.5)	
Route of administration (%)	Side port	6 (25.0)	13 (33.3)	0.578^c^
	Main route	18 (75.0)	26 (66.7)	
Drug formulation (%)	Branded	18 (75.0)	34 (87.2)	0.307^c^
	Generic	6 (25.0)	5 (12.8)	
History of diabetes mellitus (%)	Yes	4 (16.7)	6 (15.4)	1^c^
	No	20 (83.3)	33 (84.6)	
Dexamethasone premedication dose, mg	Mean ± SD	10.86 ± 3.00	10.92 ± 3.04	0.946 ^d^
Hot compress with hot towel (%)	Yes	8 (33.3)	6 (15.4)	0.124 ^c^
	No	16 (66.7)	33 (84.6)	

*CDDP* cisplatin, *VNR* vinorelbine. ^a^Du Bois formula, ^b^Mann–Whitney *U* test, ^c^Fisher’s exact test, ^d^Student’s *t*-test

**Table 3:** Multivariate analysis of factors associated with venous irritation

Clinical characteristic		AOR	95% CI	*p* value	VIF
Body surface area, m^2^	per 1 m^2^ decrement	70.42	1.54–3236.25	0.029^a^	1.097
Department	Surgery	1 (reference)			
	Respiratory medicine	0.72	0.20–2.58	0.619^a^	1.268
Hot compress with hot towel	Yes	1 (reference)			
	No	0.32	0.07–1.41	0.131^a^	1.360

*AOR* adjusted odds ratio, *CI* confidence interval, *VIF* variance inflation factor. ^a^Logistic regression analysis

## Discussion

In this study, a small body surface area was a statistically significant predictor of the risk of venous irritation associated with VNR. It is widely accepted that a small physique is associated with small vessel diameter, and the risk factors for extravasation, the symptoms of which are similar to those of venous irritation, include thin blood vessels [3]. Moreover, the volume of blood flow is thought to be correlated with body surface area [19], and it is possible that the risk of venous irritation in patients receiving VNR reflects a long drug residence time.

Venous irritation occurred in 24 (38.1%) of the 63 patients in this study. This incidence is higher than the incidence of 3.7–18.7% cited in the package insert [20]. Venous irritation is more common in clinical practice than in clinical trials. Indeed, other studies have reported incidences of venous irritation that are equivalent to or higher than our present results [10, 11]. Therefore, prevention of venous irritation is clinically relevant. Venous irritation in patients receiving VNR was not prevented by use of hot compress with a hot towel. Yokota et al. reported that combination of a hot pack compress and an increased amount of physiologic saline for flushing/washout attenuated the severity of venous irritation and decreased the vascular pain caused by VNR [16]. However, hot packs can cause burns, so we opted to use a hot towel instead, which is less likely to result in burns and is easier to apply. It has been reported that using a hot pack significantly increased skin surface temperature, rate of blood flow, and venous cross-sectional area [21]. That report suggested that the hot towel does not have the same heating effect as a hot pack because the towel cools more rapidly. However, these data are only focused on venous irritation, and we could not evaluate injection site reactions. Further research is needed to determine the utility of hot compress with a hot towel in preventing injection site reactions.

Yoh et al. reported that a high body mass index increased the risk of venous irritation while Yamada et al. reported that the risk was increased when a VNR dose ≥ 40 mg was administered [10, 11]. In contrast to these retrospective and inconclusive reports, we found that a small body surface area was the only statistically significant risk factor for venous irritation in these patients. Although both body surface area and body mass index are calculated from height and weight, they are different measures. Body mass index is the measure for evaluating obesity, while body surface area is the measure for evaluating the size of the physique. When administering VNR to patient with these risk factors, attention should be paid to venous irritation. In addition, a prospective trial is needed to determine the most significant of these risk factors.

There are several limitations to this study, First, the study had a retrospective and single-center design, a small sample size, and potential for patient selection bias. Second, we could not evaluate who secured venous access or the location of the puncture site. Furthermore, we reviewed only factors that were potentially associated with venous irritation, and further research is needed to investigate injection site reactions.

## Conclusions

In this study, body surface area was the only statistically significant predictor of the risk of venous irritation in patients receiving VNR. Hot compress using a hot towel did not prevent venous irritation. Prospective studies are needed to identify the most significant risk factor for venous irritation associated with VNR.

## Abbreviations

CDDP: Cisplatin
GEM: Gemcitabine
HER: Trastuzumab
VNR: Vinorelbine

## References

[1] Kuwahara T, Asanami S, Tamura T, Kaneda S (1998). Effects of pH and osmolality on phlebitic potential of infusion solutions for peripheral parenteral nutrition. J Toxicol Sci.

[2] Yamada T (2015). Pharmacological study and pharmaceutical intervention to reduce intravenous injection-induced vascular injury. Yakugaku Zasshi.

[3] Pérez Fidalgo JA, García Fabregat L, Cervantes A, Margulies A, Vidall C, Roila F; ESMO Guidelines Working Group. Management of chemotherapy extravasation: ESMO– EONS Clinical Practice Guidelines. Ann Oncol. 201;23:67–73. 10.1093/annonc/mds294 22997449

[4] Douillard JY, Tribodet H, Aubert D, Shepherd FA, Rosell R, Ding K (2010). Adjuvant cisplatin and vinorelbine for completely resected non-small cell lung cancer: subgroup analysis of the lung adjuvant cisplatin evaluation. J Thorac Oncol.

[5] Ohe Y, Ohashi Y, Kubota K, Tamura T, Nakagawa K, Negoro S (2007). Randomized phase III study of cisplatin plus irinotecan versus carboplatin plus paclitaxel, cisplatin plus gemcitabine, and cisplatin plus vinorelbine for advanced non-small-cell lung cancer: four-arm cooperative study in Japan. Ann Oncol.

[6] Gasparini G, Caffo O, Barni S, Frontini L, Testolin A, Guglielmi RB (1994). Vinorelbine is an active antiproliferative agent in pretreated advanced breast cancer patients: a phase II study. J Clin Oncol.

[7] Shukuya T, Takahashi T, Tamiya A, Ono A, Igawa S, Tsuya A (2009). Evaluation of the safety and compliance of 3-week cycles of vinorelbine on days 1 and 8 and cisplatin on day 1 as adjuvant chemotherapy in Japanese patients with completely resected pathological stage IB to IIIA non-small cell lung cancer: a retrospective study. Jpn J Clin Oncol.

[8] Yamada T, Egashira N, Imuta M, Yano T, Yamauchi Y, Watanabe H, Oishi R (2009). Role of oxidative stress in vinorelbine-induced vascular endothelial cell injury. Free Radic Biol Med.

[9] Tsai KL, Chiu TH, Tsai MH, Chen HY, Ou HC (2012). Vinorelbine-induced oxidative injury in human endothelial cells mediated by AMPK/PKC/NADPH/NF-κB pathways. Cell Biochem Biophys.

[10] Yoh K, Niho S, Goto K, Ohmatsu H, Kubota K, Kakinuma R (2004). High body mass index correlates with increased risk of venous irritation by vinorelbine infusion. Jpn J Clin Oncol.

[11] Yamada T, Egashira N, Watanabe H, Nagata K, Yano T, Nonaka T, Oishi R (2012). Decrease in the vinorelbine-induced venous irritation by pharmaceutical intervention. Support Care Cancer.

[12] Suzuki N, Saito K, Yokosawa D, Shimoyama T, Katagiri Y, Arao T (2010). The effect of dexamethasone administered after vinorelbine therapy for prevention of vinorelbine-induced phlebitis. J Jpn Soc Hosp Pharm.

[13] Asako E, Maruyama T, Yamada M, Yoshizaki N, Tsujimura H (2008). Bolus injection of vinorelbine reduces incidence of local venous toxicity in patients with non-small cell lung cancer and breast cancer. Gan To Kagaku Ryoho.

[14] Nakayama S, Matsubara N, Sakai T, Aso N (2002). The incidence of phlebitis in the patients administrated vinorelbine by intravenous bolus injection--a retrospective study. Gan To Kagaku Ryoho..

[15] Yoh K, Niho S, Goto K, Ohmatsu H, Kubota K, Kakinuma R (2007). Randomized trial of drip infusion versus bolus injection of vinorelbine for the control of local venous toxicity. Lung Cancer.

[16] Yokota Y, Suzuki T, Narahashi T, Takizawa J, Kojima M, Shimada R (2008). Prevention of phlebitis caused by vinorelbine chemotherapy in outpatients with breast cancer. Gan To Kagaku Ryoho..

[17] Hayashi M, Ohnishi C, Sugimura H, Miyazawa K, Yamatani A, Funaki H (2013). Incidence of injection site reactions induced by vinorelbine and prevention with hot compresses. Iyakuhin Johogaku.

[18] Kanda Y (2013). Investigation of the freely available easy-to-use software 'EZR' for medical statistics. Bone Marrow Transplant.

[19] Kawashima Y (1976). Taigai Junkan no Seiri [physiology of extracorporeal circulation]. Jinko Zouki.

[20] Navelbine (vinorelbine) Package Insert (2016) Kyowa Hakko Kirin Co., Ltd.

[21] Sasaki S, Ichimura M, Murakami N, Matsumura Y, Masaharu M, Tetsuya O (2014). Effect of hot compress on superficial vein of forearm for venipuncture. Jpn J Nurs Art Sci.

